# Inhibition of GSK-3β Enhances Osteoblast Differentiation of Human Mesenchymal Stem Cells through Wnt Signalling Overexpressing Runx2

**DOI:** 10.3390/ijms24087164

**Published:** 2023-04-12

**Authors:** Nihal AlMuraikhi, Sarah Binhamdan, Hanouf Alaskar, Amal Alotaibi, Sumaiya Tareen, Manikandan Muthurangan, Musaad Alfayez

**Affiliations:** Stem Cell Unit, Department of Anatomy, College of Medicine, King Saud University, Riyadh 11461, Saudi Arabia

**Keywords:** GSK-3β inhibition, osteoblast differentiation, human mesenchymal stem cells, Wnt, Runx2

## Abstract

Small-molecule-inhibitor-based bone differentiation has been recently exploited as a novel approach to regulating osteogenesis-related signaling pathways. In this study, we identified 1-Azakenpaullone, a highly selective inhibitor of glycogen synthase kinase-3β (GSK-3β), as a powerful inducer of osteoblastic differentiation and mineralization of human mesenchymal stem cells (MSCs). GSK-3β is a serine-threonine protein kinase that plays a major role in different disease development. GSK-3β is a key regulator of Runx2 activity in osteoblastic formation. We evaluated alkaline phosphatase activity and staining assays to assess osteoblast differentiation and Alizarin Red staining to assess the mineralization of cultured human MSCs. Gene expression profiling was assessed using an Agilent microarray platform, and bioinformatics were performed using Ingenuity Pathway Analysis software. Human MSCs treated with 1-Azakenpaullone showed higher ALP activity, increased in vitro mineralized matrix formation, and the upregulation of osteoblast-specific marker gene expression. Global gene expression profiling of 1-Azakenpaullone-treated human MSCs identified 1750 upregulated and 2171 downregulated mRNA transcripts compared to control cells. It also suggested possible changes in various signaling pathways, including Wnt, TGFβ, and Hedgehog. Further bioinformatics analysis employing Ingenuity Pathway Analysis recognized significant enrichment in the 1-Azakenpaullone-treated cells of genetic networks involved in CAMP, PI3K (Complex), P38 MAPK, and HIF1A signaling and functional categories associated with connective tissue development. Our results suggest that 1-Azakenpaullone significantly induced the osteoblastic differentiation and mineralization of human MSCs mediated by the activation of Wnt signaling and the nuclear accumulation of β-catenin, leading to the upregulation of Runx2, a key transcription factor that ultimately promotes the expression of osteoblast-specific genes. Thus, 1-Azakenpaullone could be used as an osteo-promotor factor in bone tissue engineering.

## 1. Introduction

Glycogen synthase kinase-3 (GSK-3) is a member of the serine-threonine protein kinase family and comprises two isoforms, GSK-3α and GSK-3β [1,2,3,4]. It plays regulatory roles in many physiological processes, including cell proliferation, stemness, and differentiation [1,3,4], and integrates with key intracellular signaling pathways mainly Wnt/β-catenin [1,3,4,5,6], BMP/Smad [1,3], Hedgehog [1], mTOR [3], and PI3K/Akt signaling [1]. In addition to the potential therapeutic approach of GSK-3β [2] in chronic diseases, including cancer [2,7], osteosarcoma [8], leukemia [9,10], rhabdomyosarcoma [5], type 2 diabetes mellitus [11,12], neurodegenerative diseases [13], and inflammation [10,14], GSK-3β has been identified as a negative regulator of a key inducer of bone formation, canonical Wnt/β-catenin [5,6].

Mesenchymal stem cells (MSCs) are multipotent adult stem cells that exist in multiple tissues, such as bone marrow, the umbilical cord, and adipose tissue [15,16]. MSCs can self-renew and differentiate into different cell types, including bone osteoblast cells [15,16]. The osteoblastic differentiation of human MSCs comprises complicated processes controlled by various signaling pathways, including Wnt [17,18], Tankyrase [19], JAK-STAT signaling [20,21], Hedgehog signaling [22], TGFβ [23], BMP/Smad [24], PI3K/Akt/mTOR [25], and Notch signaling [26]. However, their relative involvement on osteoblastic differentiation must be further investigated. Small-molecule inhibitors have been widely exploited as biochemical tools to help investigate the involvement of different signaling pathways in osteoblastic differentiation, which may reflect on therapeutic findings [19,20,21,22,26].

In this study, we identified a small molecule, 1-Azakenpaullone, a highly selective inhibitor of GSK-3β [27], as a powerful inducer of osteoblastic differentiation and mineralization in human MSCs. It may function as an osteo-promotor factor in bone tissue engineering. Our investigation was tailed with global gene expression profiling of human MSCs treated with 1-Azakenpaullone; we detected significant enrichment of many signaling pathways associated with osteoblastic differentiation, including Wnt, Hedgehog, and TGFβR, in addition to a significant upregulation in a number of genes essential for bone repair and mineralization, confirming the consequential effect of 1-Azakenpaullone in enhancing bone formation. Moreover, using human functional annotations and network databases, a bioinformatics analysis of the signaling networks regulated in 1-Azakenpaullon-treated human MSCs confirmed a number of activated networks in the upstream analysis and a significant increase in the gene expression of tissue development and the cellular development functional category.

## 2. Results

### 2.1. 1-Azakenpaullone Supports the Proliferation of Human MSCs

**Figure 1:**
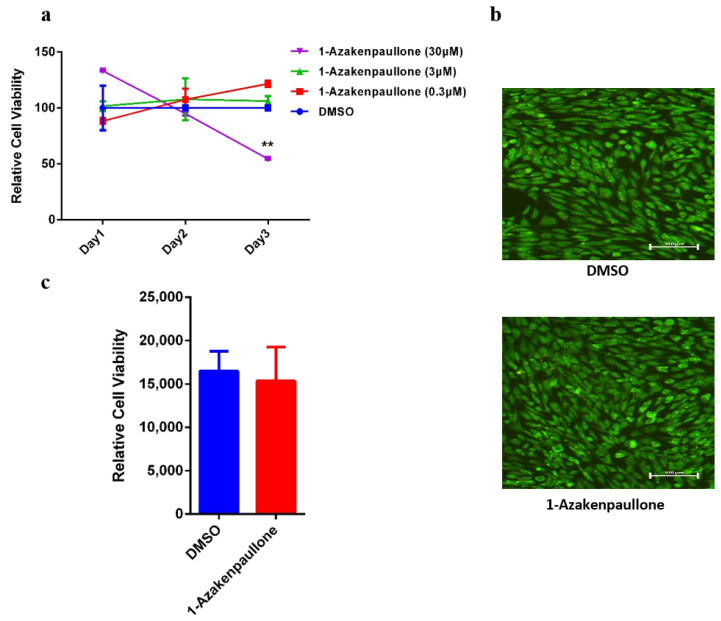
Effects of 1-Azakenpaullone treatment on the growth of human MSCs and GSK-3 signaling pathway. (**a**) Dose–response proliferation curve of human MSCs to different doses of 1-Azakenpaullone treatment, as indicated in the graph, versus DMSO-treated control cells, as measured by cell viability over 3 days. (**b**) Representative live/dead fluorescence images of 1-Azakenpaullone-treated human MSCs (3.0 µM) versus DMSO-treated control cells on day 3 after exposure. Photomicrographs magnification ×20. Cells were stained with AO/EtBr to detect apoptotic (cells with green condensed chromatin) and necrotic cells (red). (**c**) Assay for cell viability using Alamar Blue assay in 1-Azakenpaullone-treated human MSCs (3.0 µM) versus DMSO-treated control cells on day 10 post osteoblastic differentiation. Data are presented as mean ± SEM (*n* = 15); ** *p* < 0.005; DMSO: dimethyl sulfoxide.

The 1-Azakenpaullone was initially recognized as a potent inducer of osteoblastic differentiation in human MSCs, based on a functional small-molecule library screening of several small-molecule inhibitors with diverse effects, using ALP activity as a read-out in which the initial screening was conducted at a concentration of 3 μM [20]. For further investigation, we tested the dose response at a logarithmic scale, and a dose–response proliferation curve was performed on human MSCs treated with 1-Azakenpaullone at different concentrations: 0.3, 3, and 30 nM. The relative proliferation at day 1, 2, and 3 was plotted (Figure 1a). There was no significant effect of 1-Azakenpaullone on proliferation at day 1, 2, and 3 at doses of 0.3 and 3 μM. However, 30 μM 1-Azakenpaullone decreased the proliferation of human MSC cells on day 3. In addition, a live/dead assay was performed on day 3 after the cells were exposed to 1-Azakenpaullone (3 µM). This assay demonstrated a minute percentage of cell death (apoptosis and necrosis) in the 1-Azakenpaullone-treated human MSCs compared to the DMSO-vehicle treated control cells (Figure 1b). Moreover, 1-Azakenpaullone did not demonstrate any significant effect on the viability of human MSCs on day 10 of osteoblastic differentiation (Figure 1c).

### 2.2. 1-Azakenpaullone Enhances Osteoblast Differentiation of Human MSCs

**Figure 2:**
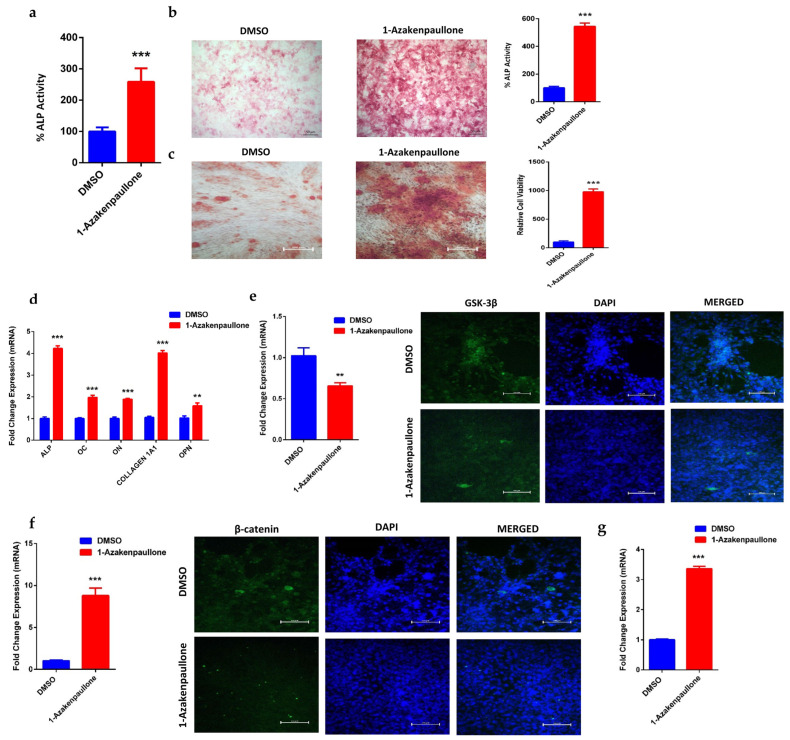
Effects of 1-Azakenpaullone treatment on the on the osteoblastic differentiation, mineralization, and gene expression of human MSCs. (**a**) Quantification of ALP activity in 1-Azakenpaullone-treated human MSCs (3.0 µM) versus DMSO-treated control cells on day 10 post osteoblastic differentiation. Data are presented as mean percentage ALP activity ± SEM (*n* = 15). (**b**) Representative alkaline phosphatase (ALP) staining of 1-Azakenpaullone-treated human MSCs (3.0 µM) versus DMSO-treated control cells on day 10 post osteoblastic differentiation; percentage of ALP staining was quantified. Photomicrographs magnification ×5. (**c**) Cytochemical staining for mineralized matrix formation using Alizarin red stained on day 14 post osteoblastic differentiation in the absence (left panel) or presence (right panel) of 1-Azakenpaullone (3.0 µM); percentage of Alizarin red staining was quantified. Photomicrograph magnification ×10. (**d**) Quantitative RT-PCR analysis for gene expression of ALP, OC, ON, COL1A1, and OPN in human MSCs on day 10 post osteoblast differentiation in the absence (blue) or presence (red) of 1-Azakenpaullone (3.0 µM). (**e**) Expression of GSK-3β in human MSCs treated with 1-Azakenpaullone (3.0 μM) for 48 h in quantitative RT-PCR analysis the absence (blue) or presence (red) of 1-Azakenpaullone (3.0 µM) for gene expression and immunocytochemistry for protein expression, scale bar: 100 μM. (**f**) Expression of β-catenin in human MSCs treated with 1-Azakenpaullone (3.0 μM) on day 10 post osteoblast differentiation in quantitative RT-PCR analysis for gene expression the absence (blue) or presence (red) of 1-Azakenpaullone (3.0 µM) and immunocytochemistry for protein expression, scale bar: 100 μM. (**g**) Quantitative RT-PCR analysis for gene expression of Runx2 in human MSCs on day 10 post osteoblast differentiation in the absence (blue) or presence (red) of 1-Azakenpaullone (3.0 µM. Gene expression was normalized to GAPDH. Data are presented as mean fold change ± SEM (*n* = 6) from two independent experiments; ** *p* < 0.005; *** *p* < 0.0005. ALP: alkaline phosphatase; OC: osteocalcin; ON: osteonectin; COL1A1: collagen type I alpha-1; OPN: osteopontin; GSK-3β: glycogen synthase kinase-3β; β-catenin: catenin beta-1; Runx2: runt-related transcription factor 2; DMSO: dimethyl sulfoxide.

Human MSCs treated with 1-Azakenpaullone (3 µM) showed a significant increase in ALP cytochemical staining intensity and ALP activity measurement compared to DMSO-vehicle treated control cells (Figure 2a,b). In addition, human MSCs exposed to 1-Azakenpaullone (3 µM) demonstrated an increase in mineralized matrix formation, verified by Alizarin red staining, compared to vehicle-treated control cells (Figure 2c). Moreover, human MSCs exposed to 1-Azakenpaullone (3 µM) significantly upregulated gene expression of osteoblast-specific marker genes including: ALP, OC, ON, COL1A1, and OPN (Figure 2d). Furthermore, to confirm that 1-Azakenpaullone targeted the GSK-3 signaling pathway by a selective inhibition of GSK-3β, human MSCs were treated with 1-Azakenpaullone at the same concentration (3 µM) and, 48 h later, the gene expression of GSK-3β was assessed using qRT-PCR and confirmed by immunocytochemistry for GSK-3β protein expression. The data presented in Figure 2e demonstrate a significant reduction in the GSK-3 signaling pathway, as evidenced by the suppression of GSK-3β expression. These data suggest that 1-Azakenpaullone might have enhancement effects on osteoblastic differentiation through the inhibition of the GSK-3 signaling pathway.

### 2.3. 1-Azakenpaullone Promotes Osteoblastic Differentiation of Human MSCs via Accumulation of β-Catenin, Which Upregulates the Expression of Runx2

Previous studies have reported that GSK-3β inhibition is associated with an increased nuclear accumulation of β-catenin and the upregulation of Runx2 [28]. Thus, we examined the gene expression of β-catenin, and nuclear localization was confirmed by the protein expression of β-catenin and to Runx2 in human MSCs. Treatment with 1-Azakenpaullone (3 µM) induced a significant upregulation in the gene expression and nuclear localization of β-catenin (Figure 2f), as well as upregulation in gene expression of Runx2 (Figure 2g), in human MSCs compared to DMSO-treated control cells, as determined on day 10 of osteoblastic differentiation. Our results suggested that the inhibition of GSK-3β by 1-Azakenpaullone might have activated Wnt signaling, leading to the accumulation of β-catenin, which subsequently promotes osteoblastic differentiation by upregulating the expression of osteoblast-specific genes, mainly Runx2.

### 2.4. Global Gene Expression Suggests Several Differentially Expressed Signaling Pathways in 1-Azakenpaullone-Treated Human MSCs

**Figure 3:**
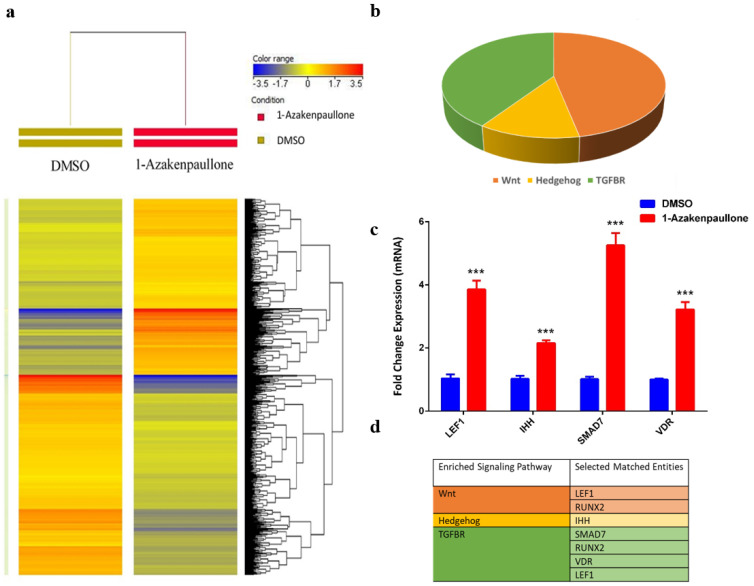
1-Azakenpaullon enhances expression of multiple signaling pathways in human MSCs during osteoblastic differentiation. (**a**) Heat map and unsupervised hierarchical clustering performed on differentially expressed genes during osteoblastic differentiation of 1-Azakenpaullon-treated human MSCs versus DMSO-treated control cells. (**b**) Pie chart demonstrating the distribution of selected signaling pathways enriched in the significantly upregulated genes identified in 1-Azakenpaullon-treated human MSCs versus DMSO-treated control cells. (**c**) Validation of a selected panel of upregulated genes in 1-Azakenpaullon-treated human MSCs versus DMSO-treated control using qRT-PCR. Gene expression was normalized to GAPDH. Data are presented as mean fold change  ±  SEM (*n*  =  6) from two independent experiments; *** *p*  <  0.0001. (**d**) Selected matched entities associated with the validated signaling pathways enriched in the significantly upregulated genes identified in 1-Azakenpaullon-treated human MSCs versus DMSO-treated control cells. Gene expression was normalized to GAPDH. Data are presented as mean fold change  ± SEM (*n*  =  6) from two independent experiments; *** *p*  ≤  0.0005. LEF1: lymphoid enhancer binding factor 1; IHH: Indian hedgehog homolog; SMAD7: mothers against decapentaplegic homolog 7; VDR: vitamin D receptor; DMSO: dimethyl sulfoxide.

**Figure 4:**
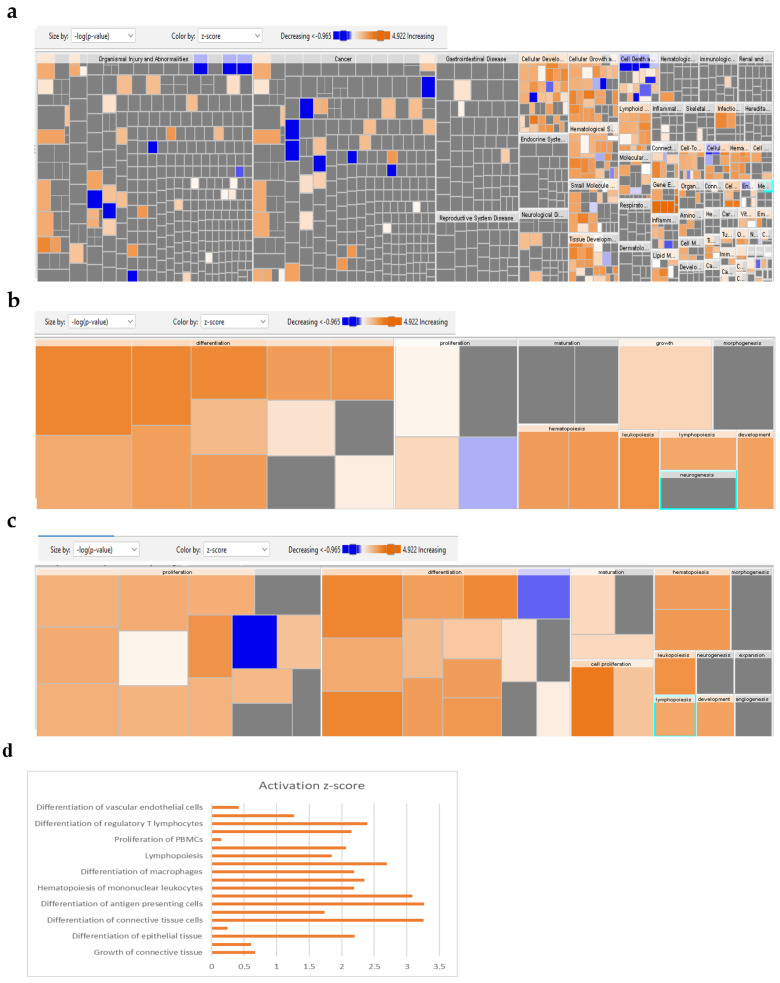

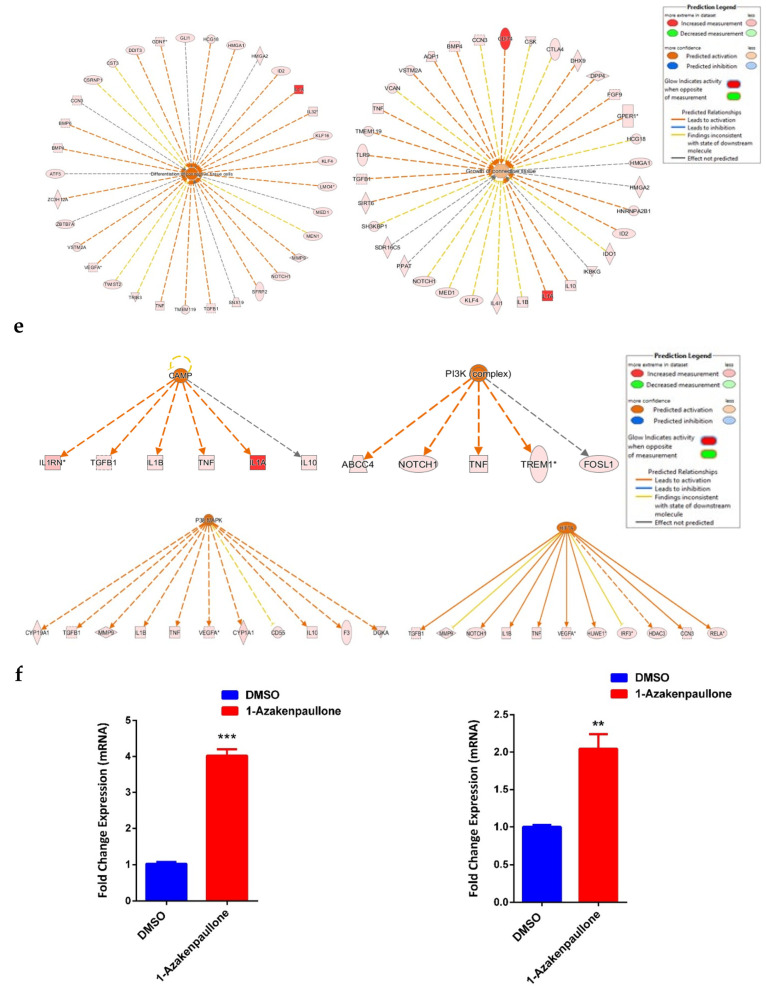
Bioinformatic analysis of signaling networks regulated in 1-Azakenpaullon-treated human MSCs. (**a**) Disease and function heat map representing activation (red) or inhibition (blue) of the specified functional and disease categories identified in the upregulated transcripts in 1-Azakenpaullone-treated human MSCs. (**b**,**c**) Heat maps demonstrating affected tissue development and cellular development functional category, respectively. (**d**) Functional annotations associated with tissue development and the cellular development functional category, based on activation z-score, with a specific illustration of differentiation and growth of connective tissues. Figure legend illustrates the interaction between molecules within the network. (**e**) Illustration of CAMP, PI3K (Complex), P38 MAPK, and HIF1A genetic networks with predicted activated state based on transcriptome data with subsequent predicted effects on TNF and TGFB1 signaling. Figure legend illustrates the interaction between molecules within the network. (**f**) Validation of predicted activation effect on the downstream effector molecules TNF and TGFB1 in 1-Azakenpaullon-treated human MSCs versus DMSO-treated control using qRT-PCR on day 10 post osteoblast differentiation in the absence (blue) or presence (red) of 1-Azakenpaullon (3.0 µM). Gene expression was normalized to GAPDH. Data are presented as mean fold change  ± SEM (*n*  =  6) from two independent experiments; ** *p* < 0.005, *** *p*  ≤  0.0005. CAMP: cathelicidin antimicrobial peptide; PI3K (Complex): phosphatidylinositol 3-kinase complex; P38 MAPK: p38 mitogen-activated protein kinases; HIF1A: hypoxia-inducible factor-1 alpha; TNF: tumor necrosis factor; TGFB1: transforming growth factor-beta; DMSO: dimethyl sulfoxide.

To identify the molecular mechanism by which 1-Azakenpaullone promotes the osteoblastic differentiation of human MSCs, we performed global gene expression profiling and a bioinformatics analysis of 1-Azakenpaullone-treated human MSCs compared to DMSO-treated controls. A heat map presented a large number of differentially expressed genes in 1-Azakenpaullone-treated cells compared to the DMSO-treated control cells (Figure 3a). We identified 1750 upregulated and 2171 downregulated genes (fold change ≥ 2.0; *p* (Corr) <0.05). A pathway analysis of the upregulated genes revealed many differentially regulated signaling pathways known to be highly associated with osteoblastic differentiation, including Wnt, Hedgehog, and TGFβR signaling (Figure 3b,d). A number of genes from the differentially regulated signaling pathways (LEF1, IHH, SMAD7, and VDR) were selected for a validation using qRT-PCR, which was consistent with the microarray data (Figure 3c,d). We next determined the enriched functional categories and molecular signaling networks controlled by 1-Azakenpaullone during the osteoblastic differentiation of human MSCs. Upregulated genes were analyzed using human functional annotations and network databases, which showed a significant increase in the gene expression of tissue development and cellular development functional category based on the activation z-score, including the growth and differentiation of connective tissue (Figure 4a–d). Additional upstream analysis showed a number of activated networks, including CAMP, PI3K (complex), P38 MAPK, and HIF1A, that shared a subsequent upregulation of downstream effector molecules TNF and TGFB1 (Figure 4e). The predicted activated networks were validated for both TNF and TGFB1 activation using the qRT-PCR, which was in agreement with the predated findings (Figure 4f). Our data suggest that 1-Azakenpaullone controls a number of signaling networks beyond GSK-3β signaling to improve the osteoblastic differentiation of human MSCs.

## 3. Discussion

Mesenchymal stem cells (MSCs) are multipotent stem cells that reside in multiple tissues, including bone marrow, and have the ability to differentiate into different cell types, including bone osteoblast cells [15,16]. The differentiation of MSCs into osteoblast cells is a complicated processes controlled by many molecular signaling pathways with different involvements that are yet to be studied [1,19,20,21,22,26]. Small-molecule inhibitors have been extensively used for this purpose [3,19,20,21,22,23,26].

Among the family of serine/threonine protein kinases, GSK-3 has proven to be a central player in the core molecular signaling of different chronic diseases, including metabolic diseases, neurological diseases, and cancer [2], and therefore emerged as a potential drug target [29]. GSK-3 consists of two isoforms, GSK-3α and GSK-3β [1,2,3,4]. Here, we identified 1-Azakenpaullone, a highly selective inhibitor of GSK-3β, as an effective inducer of the osteoblastic differentiation and mineralization of human MSCs which may be helpful in bone tissue engineering.

We demonstrated that 1-Azakenpaullone addition enhanced osteoblastic differentiation and in vitro mineral deposition, as confirmed by an increase in alkaline phosphatase activity, alizarin red staining, and the upregulation of osteoblast-specific gene expression. However, there is a lack of correlation between mRNA expression and protein level due to the post-translational modifications. Thus, it is preferred to assess the protein expression using Western blotting for further confirmation. Global gene expression profiling of human MSCs treated with 1-Azakenpaullone detected significant enrichment of many signaling pathways associated with osteoblastic differentiation, including Wnt [17], Hedgehog [22], and TGFβR [23], in addition to significant upregulation in a number of genes essential for bone repair and mineralization, confirming the consequence effect of 1-Azakenpaullone in enhancing bone formation, including LEF1, which is known to form an interdependent network with Runx2 and other signaling pathways to regulate osteoblast proliferation and differentiation [30]. The expression of IHH, which is directly required for the osteoblastic differentiation in the endochondral skeleton [31], was also significantly increased in 1-Azakenpaullone-treated human MSCs. We identified a significant increase in the expression of SMAD7, a main regulator for bone remodeling during mammalian development [32], among the top activated signaling pathways in 1-Azakenpaullone-treated human MSCs. In addition, the expression of VDR was significantly increased in 1-Azakenpaullone-treated human MSCs; VDR is approved to increase bone mass by suppressing bone resorption [33].

GSK-3β is recognized as a negative regulator of the canonical Wnt/β-catenin, which is known to be important for bone formation [5,6,9,34,35,36]. Different studies have confirmed the central role of the Wnt signaling pathway and β-catenin, its signaling mediator, in the commitment of stem cells to the osteoblast lineage [37,38]. A degradation of β-catenin, mediated by GSK-3β protein, is regulated by upstream signaling pathways, including the Akt signaling pathway [28]. However, activation of the Akt pathway inhibits GSK-3β, leading to the accumulation and translocation of β-catenin and, ultimately, the upregulation of osteoblast-specific genes [28]. The GSK-3 signaling pathway has also proven to be highly involved in the regulation of bone formation, as the modulation of GSK-3β via chemical inhibition has been shown to affect the Wnt signaling pathway and to subsequently control the expression of several downstream target genes, leading to the promotion of osteoblast differentiation [3,4,5,11,29]. In detail, the inhibition of GSK-3β activates the Wnt signaling pathway, resulting in β-catenin accumulation in the cytoplasm [39], which translocates into the nucleus later to form a complex with the T cell factor/lymphoid enhancing factor (TCF/LEF) transcriptional factor family to regulate the expression levels of specific downstream genes, thus promoting the expression of osteoblast-specific genes, including Runx2 and OC, thereby mediating osteoblastic differentiation and bone regeneration [28,37,38,40].

Osteosarcoma is a primary bone cancer in which the Wnt/β-catenin pathway is one of the key activated pathways, and β-catenin is significantly increased in osteosarcoma cells [8,41,42]. The elevated expression of β-catenin is highly related to metastasis and poor prognosis in osteosarcoma patients [41,42]. Thus, it is proposed as a therapeutic target [42]. Moreover, as it is known that GSK-3β phosphorylate Axin-bound β-catenin inhibits the translocation of β-catenin in the nucleus, some studies suggest GSK-3β as a target for the osteosarcoma treatment [41,42], while other studies propose the inhibition of GSK-3β as a therapeutic mechanism to repress cell survival and enhance apoptosis in osteosarcoma cells [8]. Since controversial conclusions have been reported on the role of GSK-3β pathway in osteosarcoma, it is crucial to further investigate the role of GSK-3β in osteosarcoma for clinical therapeutic applications.

Previous studies reported the modulation of the GSK3β/β-catenin signaling pathway during the osteoblast differentiation of human bone marrow MSCs using different drugs, including Astragaloside-IV and Metformin [43,44]. Astragaloside-IV at 40 µM mediates the osteoblast differentiation through enhancing the expression of neuronal growth factor (NGF) [43], whereas Metformin at 100 µM mediates the differentiation partially through the AMPK signaling pathway [44]. In addition, some miRNAs, such as miR-199b-5p, were also reported to activate the osteoblast differentiation of human MSCs through GSK3β/β-catenin signaling by directly targeting GSK-3β [45]. However, the mechanism remains unclear.

These studies suggest GSK-3β is a key regulator of bone formation. Our findings validate the previously reported outcomes, as we confirmed that the inhibition of GSK-3β by 1-Azakenpaullone at 3 µM increased the expression of β-catenin and upregulated the expression of Runx2, a key transcription factor that enhanced the expression of osteoblast-specific genes, including ALP, OC, ON, COL1A1, and OPN. These genes promoted osteoblastic differentiation and mineralization, which may be caused by the activation of TCF/LEF as LEF1 expression was significantly upregulated, and the activation of Wnt signaling, a key trigger for bone formation [34]. In addition, other GSK-3 inhibitors also increased the expression of other osteoblast-specific genes such as COL1A1 and ON [46,47]. In addition, the inhibition of GSK-3β by 1-Azakenpaullone significantly enhanced the activity of different signaling pathways known to be highly associated with osteoblastic differentiation, including Wnt, Hedgehog, and TGFβR signaling. Moreover, the bioinformatic analysis of signaling networks regulated in 1-Azakenpaullon-treated human MSCs confirmed a number of activated networks in the upstream analysis, including CAMP, PI3K (complex), P38 MAPK, and HIF1A, that shared a subsequent upregulation of downstream effector molecules TNF and TGFB1.

## 4. Methods

### 4.1. Cell Culture

Throughout the study, a human MSC-TERT cell line that is a model for human bone marrow MSCs was utilized. This line was genetically modified to overexpress human telomerase reverse transcriptase gene (hTERT) [48,49]. It has similar characteristic features to human MSCs in terms of self-renewal, multi-potency, and genotype [48,49].

These cells were propagated in Dulbecco’s Modified Eagle Medium (DMEM) basal medium with 4 mM L-glutamine, 4500 mg/L D-glucose, 110 mg/L 10% sodium pyruvate, 10% fetal bovine serum (FBS), 1% penicillin–streptomycin, and 1% non-essential amino acids, as previously described [48]. Reagents were procured from Thermo Fisher, Waltham, MA, USA (https://www.thermofisher.com/ accessed on 2 December 2022). Cultures were maintained under 37 °C with 5% CO_2_ and 95% humidity.

To study cell viability, an Alamar Blue assay was used according to the manufacturer’s instructions [20]. To evaluate dose response, the cells were cultured in 96-well plates in 300 μL of the DMEM medium with a mixture of 0.3, 3, and 30 μM. The 1-Azakenpaullone was compared to the DMSO-treated control cells on days 1, 2, and 3. Readings were obtained using a BioTek Synergy II microplate reader (BioTek Inc., Winooski, VT, USA) at Ex 530 nm/Em 590 nm in fluorescent mode.

### 4.2. Osteoblastic Differentiation

For osteoblast differentiation, when cells reached 70–80% confluency, induced with osteoblastic differentiation medium (DMEM containing 10% FBS, 1% penicillin–streptomycin, and 50 mg/mL L-ascorbic acid (https://www.wako-chemicals.de/ accessed on 2 December 2022), 10 mM b-glycerophosphate (Sigma-Aldrich, St. Louis, MO, USA), 10 nM calcitriol (1a,25-dihydroxyvitamin D3; Sigma-Aldrich), and 10^−9^ M dexamethasone (Sigma-Aldrich) were used. As previously described [20], the tested cells were exposed continuously to 3 µM 1-Azakenpaullone added to the osteoblastic differentiation medium over the differentiation period. Control cells were cultured in an osteoblastic induction medium containing dimethyl sulfoxide (DMSO) as a vehicle. The stem cell signaling, small-molecule inhibitor library, including 1-Azakenpaullone, was procured from Selleckchem Inc., Houston, TX, USA (Cat. No. L2100) (https://www.selleckchem.com/ accessed on 2 December 2022).

### 4.3. Measurement of Apoptosis

To measure apoptosis, fluorescence-based staining was performed using the acridine orange/ethidium bromide (AO/EtBr) as previously described [50]. Cells exposed to 3 µM 1-Azakenpaullone and DMSO-control cells were stained with a dual fluorescent staining solution (1.0 µL) containing 100 µg/mL AO and 100 µg/mL EtBr (Sigma) on day 3 for 1 min. All conditions were achieved in biological duplicates, and images from three fields/well were taken using a Nikon Eclipse Ti fluorescence microscope (Nikon, Tokyo, Japan) and assessed using NIS-Elements BR 5.01.00 software.

### 4.4. Quantification of Alkaline Phosphatase Activity

Alkaline phosphatase (ALP) activity was quantified using a Thermo Scientific ALP activity colorimetric assay kit with some modifications, as previously described [20]. Cells were cultured in 96-well plates for osteoblastic differentiation for up to 10 days. Treated cells were washed with PBS and fixed with 3.7% formaldehyde in 90% ethanol for 30 s at room temperature. The fixative was substituted with 50 µL/well of p-nitrophenyl phosphate solution and incubated for 30 min, and the OD (optical density) was measured at 405 nm using a SpectraMax/M5 fluorescence spectrophotometer plate reader (Molecular Devices, San Jose, CA, USA). ALP enzymatic activity was then normalized to the Alamar blue OD value.

### 4.5. Alkaline Phosphatase Staining

As we previously described [20], on day 10, cells cultured in a 6-well plate in osteoblastic differentiation medium were washed and fixed in 10 mM acetone/citrate buffer at pH 4.2 for 5 min at room temperature. The fixative was substituted with Naphthol/Fast Red stain (0.2 mg/mL Naphthol AS-TR phosphate substrate) (0.417 mg/mL of Fast Red) (Sigma) and kept for 1 h at room temperature. The cells were then washed and examined under a microscope. All conditions were achieved in biological duplicates, and images from three fields/well were assessed using ImageJ software (U.S. National Institutes of Health, Bethesda, MD, USA) for quantification purposes.

### 4.6. Alizarin Red S Staining for Mineralization

As we previously described [20], on day 14 of osteoblastic differentiation, cells were washed and fixed with 4% paraformaldehyde for 10 min at room temperature. They were then stained with the 2% Alizarin Red S Staining Kit (Cat. No. 0223, ScienceCell, Research Laboratories, Carlsbad, CA, USA) for 10–20 min at room temperature. The cells were then washed and examined under a microscope. All conditions were achieved in biological duplicates, and images from three fields/well were assessed using ImageJ software (U.S. National Institutes of Health, Bethesda, MD, USA) for quantification purposes.

### 4.7. RNA Extraction and cDNA Synthesis

Total RNA was extracted from two biological duplicates on day 10 of osteoblast differentiated cells using a PureLink Kit (Cat. No.: 12183018A, Ambion by Life Technologies, Carlsbad, CA, USA), and concentrations of the extracted total RNA were quantified using NanoDrop 2000 (ThermoFisher Scientific Life Sciences). The quality of the extracted total RNA was assessed by A260/280 ratio, and all samples were >2. cDNA was synthesized with 500 ng of total RNA using a High Capacity cDNA Transcription Kit (Cat. No. 4368814. ThermoFisher Scientific Life Sciences) according to the manufacturer’s instructions, as previously described [20].

### 4.8. Quantitative Real-Time Polymerase Chain Reaction

A quantitative real-time polymerase chain reaction (qRT-PCR) was performed using appropriate primers for osteoblast differentiation (listed in Table 1) with fast SYBR Green Master mix (Cat. No. 4385612), using Applied Biosystems ViiA 7 Real-Time PCR System (ThermoFisher Scientific Life Sciences) as described in [51]. An amount of 12.5 ng of cDNA/reaction was used, and all reactions were performed in technical triplicates from biological duplicates. The annealing temperature was 60 °C, and the number of cycles was 40 cycles.

### 4.9. Immunocytochemistry

To elucidate the level of protein expression of GSK-3β and β-catenin, cells were cultured in an 8-well chamber slide with 1-Azakenpaullone and DMSO as a vehicle control. The cells were fixed with 4% paraformaldehyde (PFA) for 10 min and permeabilized with 0.2% TritonX-100 for 15 min. Serum blocking was carried out with a pool of serum containing 1% bovine serum albumin and 2% horse serum. After serum blocking, the cells were incubated with GSK-3β (Cat. No. 12456, Cell Signaling Technology, Danvers, MA, USA) and β-catenin (Cat. No. 8480, Cell Signaling Technology, Danvers, MA, USA) primary antibodies in a dilution ratio of 1:500 overnight at 4 °C in a humidified chamber. This was followed by secondary Sheep anti-Rabbit IgG (H&L), conjugated with FITC (Prod. No. AS10 1537, Agrisera, Vännäs, Sweden), and DAPI staining. Images were taken using a Nikon Eclipse Ti fluorescence microscope (Nikon).

### 4.10. Gene Expression Profiling by Microarray

An amount of 200 ng of total RNA from day 10 of osteoblastic differentiation was labeled using a low-input Quick Amp Labeling Kit (Agilent Technologies, Santa Clara, CA, USA, https://www.agilent.com/ accessed on 2 December 2022) and hybridized to the Agilent Human SurePrint G3 Human GE 8 × 60 k microarray chip as described previously [52]. Pathway and functional analyses were carried out using the Ingenuity Pathway Analysis (Ingenuity Systems, Redwood City, CA, USA, https://www.ingenuity.com/ accessed on 2 December 2022) [22,53]. Upregulated genes ≤ 2 FC (fold change) and corrected *p* value < 0.05 were chosen for analysis. Enriched network categories were algorithmically calculated according to the Z-score.

### 4.11. Statistical Analysis

Statistical analysis and graphing were carried out using Microsoft Excel 2010 and GraphPad Prism 6 software, respectively. Outcomes were displayed as the mean ± SEM from at least two independent experiments. An unpaired, two-tailed Student *t*-test or two-way ANOVA were used to compute statistical significance according to the number of variables, and *p*-values  < 0.05 were considered statistically significant.

## 5. Conclusions

This study suggests 1-Azakenpaullon, a selective inhibitor for GSK-3β, as an enhancer for the osteoblastic differentiation and mineralization of human MSCs by activating Wnt signaling and controlling Runx2 activity. Thus, it might be used as an osteo-promotor factor in bone tissue engineering.

## Figures and Tables

**Table 1:** List of SYBR Green primers used in this study.

Gene Name	Official Symbol	Accession Number	Product Length	Forward Primer	Reverse Primer
*GAPDH*	GAPDH	NM_001256799.3	81	CTGGTAAAGTGGATATTGTTGCCAT	TGGAATCATATTGGAACATGTAAACC
*ALP*	ALPL	NM_001127501.4	86	GGAACTCCTGACCCTTGACC	5′TCCTGTTCAGCTCGTACTGC3′
*OC*	BGLAP	NM_001199662.1	102	GGCAGCGAGGTAGTGAAGAG	CTCACACACCTCCCTCCTG
*ON*	SPARC	NM_001309444.2	95	GAGGAAACCGAAGAGGAGG	5′GGGGTGTTGTTCTCATCCAG3′
*RUNX2*	RUNX2	NM_001015051.4	78	GTAGATGGACCTCGGGAACC	5′GAGGCGGTCAGAGAACAAAC3′
*OPN*	SPP1	NM_000582.3	348	GGTGATGTCCTCGTCTGTA	CCAAGTAAGTCCAACGAAAG
*COL1A1*	COL1A1	NM_000088.4	52	5′GAGTGCTGTCCCGTCTGC3′	5′TTTCTTGGTCGGTGGGTG3′
*GSK-3β*	GSK3B	NM_001146156.2	106	5′GGAACTCCAACAAGGGAGCA3′	5′TTCGGGGTCGGAAGACCTTA3′
*β-catenin*	CTNNB1	NM_001330729.2	108	5′ATGGAGCCGGACAGAAAAGC3′	5′CTTGCCACTCAGGGAAGGA3′
*LEF1*	LEF1	NM_001130713.3	134	5′CTTTATCCAGGCTGGTCTGC	5′TCGTTTTCCACCATGTTTCA
*IHH*	IHH	NM_001346281.2	109	CAGCGATGTGCTCATTTTCCT	AAGGCTCTCAGCCTGTGAGG
*SMAD7*	SMAD7	NM_005904.4	95	CCCATCACCTTAGCCGACTC	TGGACAGTCTGCAGTTGGTTT
*VDR*	VDR	NM_001017535.2	87	CTCTGATAGCCTCATGCCAGG	ACCCAAAGGCTTCCCAAAGAG
*TGFB1*	TGFB1	NM_000660.7	300	5′GAAACCCACAACGAAATC3′	5′AATTTCCCCTCCACGGCT3′
*TNF*	TNF	NM_000594.4	139	5′ACTTTGGAGTGATCGGCC3′	5′GCTTGAGGGTTTGCTACAAC3′

## Data Availability

The data presented in this study are available upon request from the corresponding author.

## Abbreviations

MSCs: Mesenchymal stem cells; GSK-3: glycogen synthase kinase-3; ALP: alkaline phosphatase; DMEM: Dulbecco’s Modified Eagle’s Medium; DMSO: dimethyl sulfoxide; MSCs: mesenchymal stem cell; hTERT: human telomerase reverse transcriptase; PBS: phosphate-buffered saline; qRT-PCR: quantitative reverse transcriptase—polymerase chain reaction; BMP: bone morphogenetic proteins; Wnt: wingless/int1; mTOR: mammalian target of rapamycin; TGF-b: transforming growth factor-β; TNF: tumor necrosis factor; Runx2: runt-related transcription factor 2; OC: osteocalcin; ON: osteonectin; OPN: osteopontin; COL1A1: alpha-1 type I collagen; GSK-3β: glycogen synthase kinase-3β; β-catenin: catenin beta-1; LEF1: lymphoid enhancer binding factor 1; IHH: Indian hedgehog homolog; SMAD7: mothers against decapentaplegic homolog 7; VDR: vitamin D receptor; CAMP: cathelicidin antimicrobial peptide; PI3K (Complex): phosphatidylinositol 3-kinase complex; P38 MAPK: p38 mitogen-activated protein kinases; HIF1A: hypoxia-inducible factor-1alpha.
